# Daily intake of broccoli sprouts normalizes bowel habits in human healthy subjects

**DOI:** 10.3164/jcbn.17-42

**Published:** 2017-11-03

**Authors:** Akinori Yanaka

**Affiliations:** 1 Hitachi Medical Education and Research Center, University of Tsukuba Hospital, Division of Gastroenterology, Faculty of Medicine, University of Tsukuba, Tsukuba, Ibaraki 305-8575, Japan

**Keywords:** broccoli sprouts, sulforaphane, defecation, oxidative stress, constipation score system

## Abstract

Chronic oxidative stress impairs regular defecation. Sulforaphane (SFN) enhances anti-oxidant systems, ameliorating oxidative injury. SFN inhibits overgrowth of anaerobic microflora and protects small intestine from oxidative injury. We assessed whether daily intake of SFN-rich broccoli sprouts (BS) improves defecation in humans. Forty-eight subjects, with a constipation scoring system (CSS) >2 points, were assigned to either the BS group (*n* = 24) or the alfalfa sprouts (AS) group (*n* = 24), and were requested to eat 20 g daily of raw BS or AS, respectively, for 4 weeks. BS contains 4.4 mg/g sulforaphane glucosinolates (SGS), while AS contains no SGS. CSS-based questionnaires were performed to evaluate bowel habit. Stool samples were collected to evaluate intestinal microflora using a terminal restriction fragment length polymorphism flora analysis. Intervention with BS, but not AS, caused a significant decrease in the duration of attempted defecation and the total CSS score. Intervention with BS decreased the percentage of *Bifidobacterium* in the stool. These results suggest that daily intake of BS improves bowel habit in human subjects. Since BS treatment enhance antioxidant enzyme activities, these effects of BS appear to relate with the SFN-mediated modulation of the intestinal motility during exposure to oxidative stress. (UMIN Clinical Trial Registration Number: UMIN-000021207)

## Introduction

Reports indicate that worldwide, a significant number of people have defecation problems in the daily life.^(1)^ A recent study conducted after the great earthquake hit eastern Japan on March 2011 showed that chronic constipation was strongly associated with an unbalanced daily diet due to the increased psychological stress caused by the disaster.^(2)^ The persistence of chronic constipation frequently impairs the quality of life; therefore, it is important to resolve these issues by implementing lifestyle changes.

The lower gastrointestinal (GI) tract plays an important role in a variety of physiological functions, such as the secretion and absorption of water, minerals, and nutrients, as well as the excretion of stools. Extensive chronic oxidative stress has been shown to disturb defecation.^(3,4)^ Therefore, it seems reasonable to assume that anti-oxidant compounds may improve defecation, particularly in conditions associated with chronic oxidative stress. It has been reported that sulforaphane (SFN), contained in broccoli sprouts (BS), enhances *nrf2*-*keap1*-mediated anti-oxidant systems, and thereby protects cells and organs from different types of oxidative stress.^(5–8)^ Thus, it may be possible that BS-derived sulforaphane may upregulate anti-oxidant activity of intestinal cells, thereby helping preserve normal intestinal function against chronic oxidative stress. On the other hand, we have previously demonstrated that SFN exhibits anti-bacterial activity against gastric *Helicobacter pylori* (*H. pylori*) and anaerobic bacteria in the small intestinal mucosa.^(9–11)^ Moreover, recent studies have shown that some intestinal microflora, such as *Bifidobacteria*, improve defecation.^(12,13)^ Therefore, it may be also possible that SFN modulates intestinal microflora, promoting smooth defecation.

The present study was conducted to assess if daily intake of SFN-rich raw BS improves defecation in basically healthy human subjects and to determine if the potential beneficial effects of SFN on defecation are associated with upregulation of anti-oxidant enzymes of SFN and/or alteration of intestinal microbiota.

## Methods

In order to evaluate whether daily intake of BS alters bowel habits in healthy human volunteers, a placebo-controlled semi-open label intervention trial was designed. This study was approved by the ethical committee of the Hitachi General Hospital (Approval Number: 2015-63), and was registered with the University Hospital Medical Information Network in Japan (UMIN Registration Number: UMIN-000021207). The trial is registered as “Studies in the effects of dietary intake of broccoli sprouts on intestinal microflora and bowel movements in healthy human subjects.”

### Participant recruitment

**Fig. 1:**
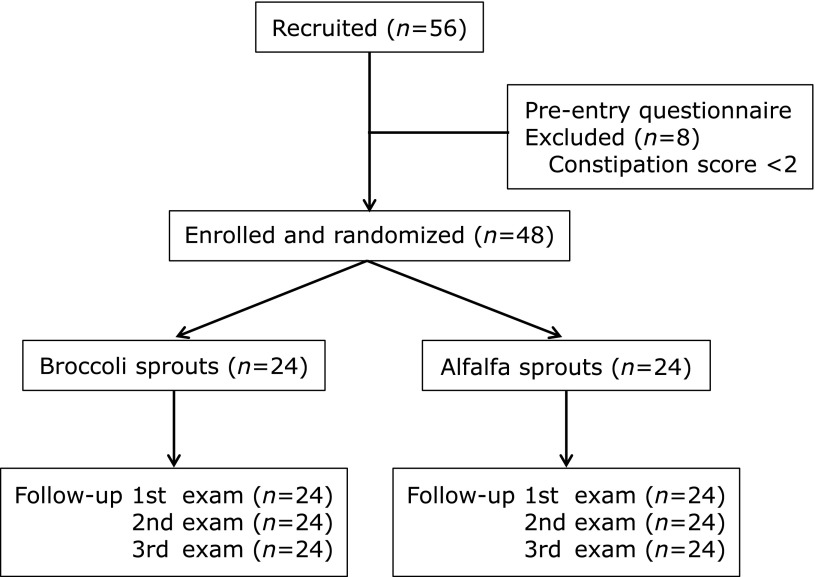
Clinical trial flow diagram on the effect of an intervention trial with broccoli or alfalfa sprouts on bowel habits in human healthy subjects. Out of a total of 56 candidates, 8 subjects with the constipation scores <2 points were excluded from the study. All remaining 48 participants completed the protocol.

Fifty-six subjects were recruited from employees of the Hitachi General Hospital. All subjects agreed to participate in this study and signed a written informed consent form. All participants completed questionnaires regarding ordinary defecation patterns, using a modified constipation scoring system (CSS).^(14)^ The original CSS consists of the following 8 factors: frequency of bowel movements, painful evacuation, incomplete evacuation, abdominal pain, duration of defecation attempt, assistance for evacuation, unsuccessful attempts of evacuation per 24 h, and duration of constipation history. In this study, we modified the original CSS, by excluding the duration of constipation history, as this score does not reflect changes in defecation after intervention. The details of the modified CSS used in this study are shown in Table 1. The following exclusion criteria were applied: subjects with CSS scores <2 points or subjects regularly using laxatives or antibiotics, as these may alter defecation patterns. A past or present history of GI disorders or disorders affecting other organs, such as liver, kidney, and endocrine organs was also included as an exclusion criterion; however, it did not apply to any subjects. Based on the results from the recruitment interview and the CSS-based pre-entry questionnaires, 8 subjects with a constipation score <2 points were excluded from the study (Fig. 1). The remaining 48 subjects consisted of 44 nurses and 4 medical technologists. All of them have been living basically healthy lives without any needs for medications or hospital/clinic visits.

### Protocol for sprouts intervention

In this study, we used alfalfa sprouts (AS) as placebo because AS contains virtually no sulforaphane glucosinolates (SGS), a precursor of SFN. BS contains 4.4 mg/g of SGS. According to the data from the manufacturer, and the Standard Tables of Food Composition in Japan (http://www.mext.go.jp/a_menu/syokuhinseibun/1365297.htm), there was no significant difference between BS and AS in the composition of almost all other nutrients, minerals, vitamins and dietary fibers, although the content of retinol and β-carotene was greater in BS than in AS (Table 2). However, according to the data from the Standard Tables of Food Composition in Japan, the total concentration of retinol and β-carotene in BS was far lower than that included in several other foods in ordinary diet.

**Fig. 2:**
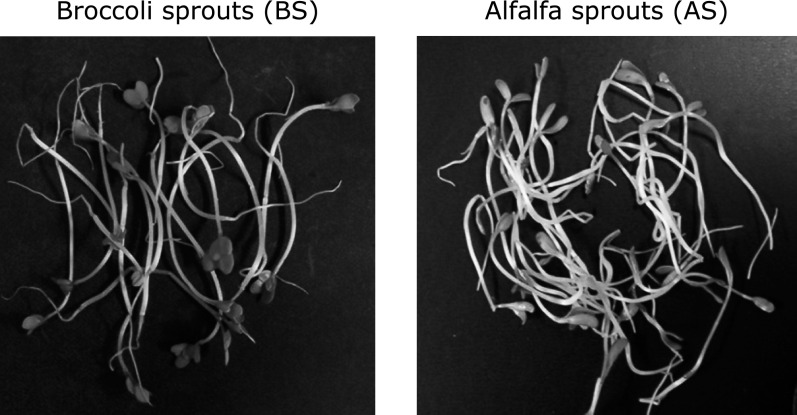
Gross appearance of broccoli sprouts and alfalfa sprouts. Alfalfa sprouts (AS) were used as placebo control, since they contain no sulforaphane (SFN), while broccoli sprouts (BS) contain a very high concentration of SFN. There was no large difference in other major nutrient components between the AS and BS. The gross appearance of AS resembles that of BS, although the taste of AS is not so spicy compared to that of BS.

**Fig. 3:**
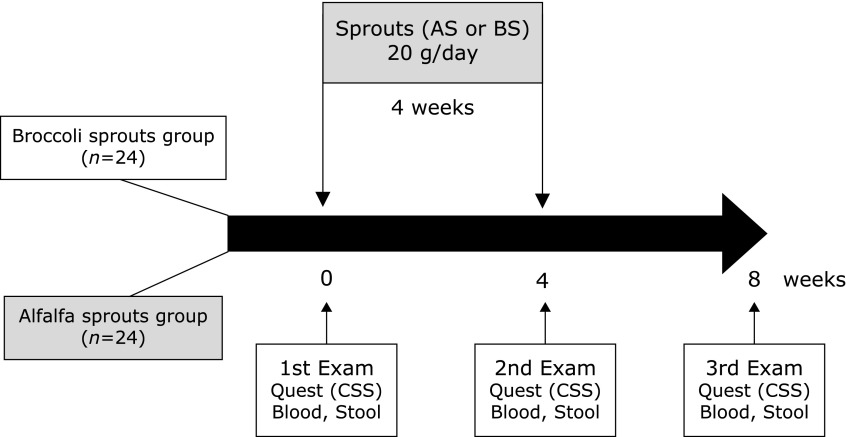
Protocol for the clinical trial on the effects of dietary broccoli or alfalfa sprouts intervention on bowel habits in healthy human subjects. Forty-eight participants were assigned to either a BS group or an AS group and were requested to consume 20 g/day of raw sprouts, every day for 4 weeks. Questionnaires, blood and stool samples were collected just before, just after and at 4 weeks after the intervention.

**Fig. 4:**
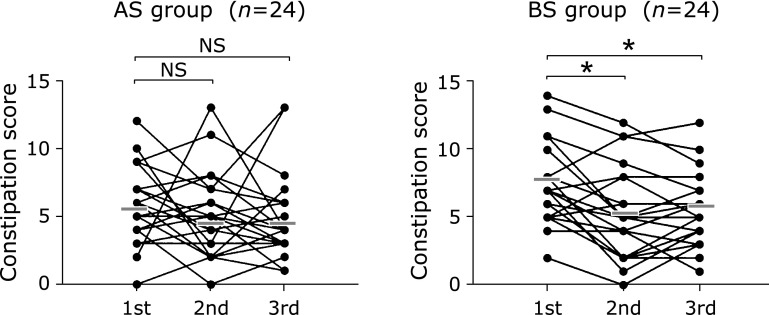
Changes in the constipation score after broccoli or alfalfa sprouts intervention. *n*; number of the subjects, Gray bars; mean values, *p<0.05, significant difference from the corresponding values at the 1st examination. NS; not significant. Questionnaires were collected on 3 occasions: immediately before the intervention (1st examination), at the end of the intervention (2nd examination), and 4 weeks after the post-trial observation period (3rd examination). The mean total constipation score decreased significantly after the 4-week intervention with BS, but not with the AS. The decreased constipation score in the BS remained low at 4 weeks after intervention cessation.

Based on the data obtained at the recruitment interview, subjects were assigned to the BS group (*n* = 24) or the AS group (*n* = 24) (Fig. 1). Allocation was conducted in order to minimize differences in age, male/female ratio, and the CSS score before the entry between the two groups. As a result, no difference was observed in age, male/female ratio, and the CSS score at the recruitment interview between the two groups; only the body mass index (BMI) in the AS group was slightly, but significantly, greater than the BMI in the BS group (Table 3). Both BS and AS were commercially available. However, in this study, all the sprouts were cultured and harvested at Murakami Farm Co. Ltd, and the fresh raw sprouts were delivered to the participants’ home or office twice a week during the treatment period. The gross appearance of AS resembles that of BS, although the taste of AS is not so spicy compared to that of BS (Fig. 2). Subjects were not informed whether they were assigned to either the BS or the AS group. To exclude the potential effects of some foods and drugs on the CSS and the laboratory data, subjects were requested to refrain from consuming cruciferous vegetables, fermented foods, laxatives, probiotics, and antibiotics, throughout the entire 10-week study period, which included the 2-week pre-trial period, the 4-week intervention period, and the 4-week post-trial observation period. During the intervention period, subjects were requested to eat either 20 g of raw BS or AS every day for 4 weeks. After the 2-week pre-trial period, stool and blood samples were collected in the morning from all participants and these were submitted with the written CSS-based questionnaires. Samples and questionnaires were collected on 3 occasions: immediately before the intervention (1st examination), at the end of the intervention (2nd examination), and 4 weeks after the post-trial observation period (3rd examination) (Fig. 3, 4 and Table 4).

### Sample analysis

Blood samples were collected to analyze complete blood count; liver, kidney and thyroid function; and glucose tolerance. Stool samples were collected to measure the amount of ammonia and to evaluate intestinal microflora. The composition of the intestinal microflora in each stool sample was evaluated using terminal restriction fragment length polymorphism flora analysis according to Nagashima’s method,^(15)^ which revealed the percentage of *Bifidobacterium*, *Lactobacillus*, *Bacteroides*, *Prevotella*, *Clostridium* [cluster IV, IX, XI, XIVa, XVIII] and other organisms. Stool and blood sample analyses were performed at Techno Suruga Lab, Shizuoka, Japan, and SRL Co., Ltd., Saitama, Japan, respectively.

### Data analysis

The student’s *t* test was used for the analysis of continuous data with a normal distribution. Non-parametric tests were used for continuous data that did not show a normal distribution and with discrete data. For non-parametrical analysis, the Wilcoxon signed-rank test and the Mann-Whitney *U* test were applied for comparison of unpaired and paired data, respectively. *P* values less than 0.05 were considered to be statistically significant.

## Results

### Effects of BS/AS intervention on modified CSS

We observed a slight increase in the constipation score only in the BS group during the pre-intervention period (Table 3 and 4), but the difference was not statistically significant (*p* = 0.104). Intervention with BS, but not AS, showed a significant decrease in the duration of defecation attempt score from 0.96 ± 0.62 to 0.58 ± 0.58 (*p* = 0.0077), suggesting that BS shortens the duration of defecation. BS consumption also resulted in a significant decrease in the total constipation score from 7.25 ± 2.83 to 5.17 ± 3.27 (*p* = 0.0017), indicating that BS promote smooth defecation. These effects of BS on the CSS persisted for 4 weeks even after cessation of the BS treatment. In contrast, AS treatment did not affect the scores during the study period (Table 4 and Fig. 4).

### Effects of BS/AS intervention on intestinal microflora and ammonia in stool samples

BS, but not AS treatment, caused a significant decrease in the percentage of *Bifidobacterium* after the 4-week treatment (*p* = 0.0498). The percentage of the *Bifidobacterium* returned to the pre-intervention level at 4 weeks after cessation of the BS treatment. There were no significant changes in the prevalence of other intestinal bacteria by either the BS or the AS treatment. Ammonia content in the stool samples did not change after the BS or the AS treatment (Table 5).

### Effects of BS/AS intervention on laboratory blood data and clinical findings

All participants tolerated the daily intake of 20 g/day of BS/AS well and were examined during the 4-week intervention period, per the protocol. No participants demonstrated clinical symptoms during the trial (Fig. 1). Both in the AS and the BS group, all laboratory data, including complete blood count; liver, kidney and thyroid function; and glucose tolerance, remained within standard ranges throughout the whole trial period (data not shown), with the only exception shown in 2 cases of the AS group, which showed mild liver dysfunction before the clinical trial, with ALT levels of 44 and 82 U/L. However, AS treatment did not worsen liver dysfunction further, suggesting that these values are not related to the effect of this intervention study. In addition, the BS group showed a small decrease in the levels of triiodothyronine (T_3_) within the physiological range immediately after the 4-week treatment (Table 6). The mean T_3_ values in the BS group returned to the initial levels after the subsequent 4-week post-trial period. No significant changes were detected in the levels of T_4_ and TSH (Table 6). No subjects showed hypothyroid signs or symptoms throughout the entire study period.

## Discussion

The present study shows that a daily intake of 20 g/day of raw BS for 4 weeks improves defecation in healthy subjects. However, this effect was not demonstrated by intake of the same amount of AS. We have confirmed that the BS used in this study contains a relatively high concentration of 4.4 mg/g SGS, while the AS contains virtually no SGS. It has been suggested that biologically inactive SGS included in the orally administrated raw BS is converted into SFN, which is the biologically active form in the intestinal lumen, probably by action of intestinal bacterial myrosinase.^(16)^ As there was no significant difference between BS and AS in the composition of all other nutrients, minerals, and vitamins (Table 2), we postulated that improvement in defecation after BS intake was caused by SFN included in the BS. Our previous clinical trials showed that oral intake of BS, which contains 128 mg SGS, up-regulates heme oxygenase-1 (HO-1) expression in blood lymphocytes in human healthy subjects.^(9)^ We have also shown that oral intake of 30 mg SGS supplement up-regulates expression of HO-1 and NAD(P)H quinone dehydrogenase 1 in human healthy subjects.^(17)^ Since the amount of SGS administered to the BS group in this study was 88 mg/day, it seems plausible that HO-1 was up-regulated in the BS group in this study.

Human GI tracts are ordinarily exposed with various noxious agents, such as *H. pylori*, NSAIDs, and ischemia.^(18,19)^ All of these factors load oxidative stress to GI tracts by generating free radicals, thereby cause various types of GI disorders, such as inflammations, ulcers, cancers, and functional GI disorders. However, during exposure to oxidative stresses, GI tracts show adaptive protection by up-regulating the *nrf2*-mediated antioxidant enzymes, such as HO-1, which scavenge free radicals.^(20)^ A number of previous studies have shown that various types of antioxidant agents strengthen antioxidant system, thereby protect GI tract from oxidative stresses.^(9–11,21,22)^ For example, ghrelin,^(21)^ a gut hormone highly expressed in gastric mucosa, protects gastric mucosa from oxidative injury by scavenging free radicals. Rebamipide, a mucosal protective agent known to scavenge free radicals, accelerates healing of artificially generated human gastric ulcers.^(22)^ Furthermore, we have previously shown that SFN enhances *nrf2*-*keap1*-mediated antioxidant systems, thereby ameliorates *H. pylori*-induced gastritis and prevents NSAIDs-induced ulcers in small intestine.^(9–11)^ Therefore, we postulated that up-regulation of antioxidant enzymes by SFN during the BS treatment contributed to maintenance of the normal intestinal motility during exposure to oxidative stress in daily lives, which in turn resulted in improved defecation by the BS treatment.

We initially assumed that SGS may affect intestinal microflora, thereby improving defecation, as our previous studies showed that SGS inhibits colonization of gastric mucosal *H. pylori* and suppresses mucosal invasion of anaerobic bacteria into the small intestinal mucosa.^(9–11)^ In the present study, however, intake of BS reduced the percentage of *Bifidobacterium* organisms, which have been regarded as the beneficial bacteria improving defecation. Furthermore, BS intake did not affect stool ammonia content, suggesting that BS treatment did not influence ammonia-producing bacteria. Therefore, we believe that the beneficial effects of BS treatment on defecation were not related to the changes in the intestinal microflora, but were caused as a result of the up-regulation of anti-oxidant enzyme activities by SFN included in BS.

It is possible that other compounds contained in the BS may also contribute to the improvement of defecation in the BS group. According to the data in Table 2, dietary fiber, particularly insoluble dietary fiber, may contribute to the beneficial effects of BS on defecation, as consumption of dietary fiber has been shown to increase frequency of defecation,^(23)^ and the amount of insoluble dietary fiber in 20 g of BS is 0.36 g, which is greater than 0.26 g contained in 20 g AS. However, the daily amount of insoluble dietary fiber contained in ordinary diets is between 10 and 15 g.^(24)^ Therefore, it is likely that the difference of 0.10 g in the amount of insoluble dietary fiber between 20 g of BS and 20 g of AS, would be masked by ordinary diet. The data in Table 2 also show that the concentrations of retinol and β-carotene are far greater in BS than in AS. However, there are a number of other foods in ordinary diet, which contain even higher amounts of retinol or β-carotene. According to the Standard Tables of Food Composition in Japan, liver, eggs and butter are rich in retinol, and carrots, spinach and pumpkins are rich in β-carotene. For example, the total amount of retinol in 50 g liver + 50 g eggs + 10 g butter is estimated to be 7,150 µg, while the amount of retinol included in 20 g BS is only 15.6 µg. Similarly, the total amount of β-carotene in 50 g carrots + 50 g spinach + 100 g pumpkins is estimated to be 10,200 µg, while the amount of β-carotene in 20 g BS is only 186 µg. Thus, it is likely that the small difference in the amount of retinol or β-carotene between 20 g BS and 20 g AS would be masked by the large amount of those compounds in other foods of the diet.

We should consider also the amount of SGS included in the ordinary diet during the intervention period. We estimate that the total SFN intake during the trial was far greater in the BS group than in the AS group. Cruciferous vegetables, such as cabbage and Japanese radish, are rich in SGS. However, the amount of SGS in cabbage and Japanese radish has been reported to be only about 10–15 mg/100 g.^(25)^ In contrast, BS used in this study contain 440 mg of SGS/100 g. Since we requested all the participants to refrain from taking large amounts of cruciferous vegetables, we assume that the total SFN intake was greater in the BS group than in the AS group during the trial. This assumption is strongly supported by our previous report,^(9)^ in which we have demonstrated that urinary excretion of SFN metabolites is far greater in the BS group compared to the AS group during the clinical trial performed using the same protocol as this study.

In the present study, all participants well tolerated the daily intake of 20 g/day of BS/AS and completed the study, per protocol, with no dropouts. No subjects demonstrated clinical symptoms or abnormal laboratory data following the intake of the BS or the AS throughout the study period. The only change detected after the 4-week BS treatment was a slight decrease in T_3_ levels. However, the absolute T_3_ data recorded in this study remained within the standard range during the 4 weeks of intervention with BS. The T_3_ levels returned to the initial values at 4 weeks after cessation of the BS treatment. Furthermore, there were no significant changes in the levels of T_4_ or TSH. No subjects presented with clinical dysfunction associated with hypothyroidism after BS treatment. It has been shown that brassica vegetables contain goitrin, which has been known to inhibit thyroid functions. An old animal study shows that intake of large amount of brussels sprouts, which contains highest amount of goitrin among brassica vegetables, suppresses thyroid function in rats.^(26)^ However, a clinical trial conducted later showed that daily consumption of 150 g of brussels sprouts for 4 weeks did not impair thyroid function in humans.^(27)^ A recent report have estimated that daily intake of 100 g brassica vegetables does not increase the plasma goitrin level to impair thyroid functions in humans.^(28)^ Although there have been no reports examining the direct effect of BS on human thyroid functions, the present data clearly show that daily intake of 20 g BS for 4 weeks is safe for normal healthy human subjects. Further research is required to determine the maximal safe amount and the longest safe duration of daily BS intake.

### Study Limitations

First, although subjects were not officially informed whether they were assigned to either the BS or the AS group, some participants seemed be able to differentiate BS and AS, from the difference in the taste and the shape of the BS and AS. Second, although there were restrictions on the intake of some foods and drugs, which may affect the data, there were no other restrictions on daily diet. In fact, we observed a slight increase in the constipation score in the BS group after 2 weeks of dietary restriction in the pre-intervention period. Therefore, it is possible that the data in this study were affected by changes in dietary components during the trial period, and/or by differences in compositions of daily diet among the participants. Third, although the present study suggests that SFN included in BS plays an important role in the improvement of defecation in human subjects, we do not show direct evidence for SFN alone in this effect. Thus, in the near future, we need to perform a new clinical trial using pure SFN compound, instead of using BS. We believe, however, that the most important message from the present study is that we were able to control defecation to some extent by a dietary approach alone, without using SFN supplements or other medications.

## Conclusion

In conclusion, the present study demonstrates that intake of 20 g of BS, but not AS, significantly enhances defecation with no side effects in healthy human subjects, and these beneficial effects appear to be induced by the protective effects of SFN in the BS on GI function against chronic oxidative stress from daily life.

## Figures and Tables

**Table 1:** Constipation scoring system

Score	0	1	2	3	4
Frequency of bowel movements	1–2 times/1–2 days	2 times/week	Once/week	Less than once/week	Less than once/month
Difficulty: painful evacuation effort	Never	Rarely	Sometimes	Usually	Always
Completeness: feeling incomplete evacuation	Never	Rarely	Sometimes	Usually	Always
Pain: abdominal pain	Never	Rarely	Sometimes	Usually	Always
Time: minutes in lavatory per attempt	Less than 5	5–10	10–20	20–30	More than 30
Assistance: type of assistance	Without assistance	Stimulative laxatives	Digital assstance or enema	—	—
Failure: unsuccessful attempts for evacuation per 24 h	Never	1–3	3–6	6–9	More than 9

**Table 2:** Major nutrients in broccoli sprouts and alfalfa sprouts

Sulforaphane glucosinolates (SGS)			Broccoli sprouts	Alfalfa sprouts
		mg	440	0
Energy		kcal	41	12
Water		g	87.4	96
Protein		g	4.7	1.6
Lipids		g	0.7	0.1
Carbohydrates		g	6.6	2
Minerals		g	0.6	0.3
Sodium		mg	3	7
Potassium		mg	105	43
Calcium		mg	66	14
Magnesium		mg	43	13
Phosphorus		mg	121	37
Iron		mg	1.1	0.5
Zinc		mg	0.6	0.4
Copper		mg	0.04	0.09
Manganese		mg	0.55	0.1
β-carotene		µg	930	56
Retinol		µg	78	5

Vitamins	D	µg	—	—
	E	mg	2.5	1.9
	K	µg	125	47
	B_1_	mg	0.16	0.07
	B_2_	mg	0.17	0.09
	Nyacin	mg	2.6	0.2
	B_6_	mg	0.3	0.1
	B_12_	µg	0	0
	Folic acid	µg	170	56
	Pantothenic acid	mg	1.04	0.46
	C	mg	80	5

Dietary fibers	Water soluble	g	0.3	0.1
	Water insoluble	g	1.8	1.3
	
	Total amount	g	2.1	1.4

**Table 3:** Subject profile

	Alfalfa sprouts	Broccoli sprouts
# of subjects	24	24
Sex (M:F)	2:22	2:22
Age	35.0 ± 8.28	35.0 ± 8.20
Height	160 ± 6.63	162 ± 6.72
Weight	60.3 ± 12.4	55.9 ± 7.29
BMI	23.5 ± 5.00	21.4 ± 2.86*
Constipation score	6.75 ± 2.89	6.54 ± 2.81

Values are expressed as mean ± SD. *p<0.05: Significant difference from the corresponding value in AS group.

**Table 4:** Changes in bowel habits after AS/BS intervention

		1st	2nd	3rd
Alfalfa sprout group (*n* = 24)				
Constipation scoring system				
	Frequency of bowel movements	0.50 ± 0.66	0.50 ± 0.98	0.63 ± 1.10
	Painful evacuation	1.42 ± 0.83	1.08 ± 1.14	1.00 ± 0.93
	Incomplete evacuation	1.63 ± 1.06	1.29 ± 0.81	1.17 ± 1.09
	Abdominal pain	1.00 ± 0.88	1.08 ± 0.83	1.17 ± 0.96
	Duration per attempt	0.58 ± 0.65	0.46 ± 0.66	0.42 ± 0.58
	Assistance for evacuation	0.04 ± 0.20	0.04 ± 0.20	0.08 ± 0.28
	Unsuccessful evacuation	0.58 ± 0.50	0.42 ± 0.50	0.38 ± 0.49
	
	Total constipation score	5.75 ± 2.71	4.83 ± 3.07	4.83 ± 3.10

Broccoli sprout group (*n* = 24)				
Constipation scoring system				
	Frequency of bowel movements	0.50 ± 1.02	0.33 ± 0.92	0.42 ± 0.78
	Painful evacuation	1.63 ± 0.97	1.13 ± 0.95	1.17 ± 0.92
	Incomplete evacuation	1.71 ± 1.23	1.17 ± 1.20	1.25 ± 0.94
	Abdominal pain	1.63 ± 1.10	1.33 ± 1.09	1.46 ± 0.93
	Duration per attempt	0.96 ± 0.62	0.58 ± 0.58*	0.63 ± 0.58*
	Assistance for evacuation	0.13 ± 0.34	0.04 ± 0.20	0.08 ± 0.28
	Unsuccessful evacuation	0.71 ± 0.55	0.58 ± 0.88	0.46 ± 0.51
	
	Total constipation score	7.25 ± 2.83	5.17 ± 3.27*	5.46 ± 2.57*

Values are expressed as mean ± SD. *p<0.05: Significant difference from the corresponding value at 1st examination.

**Table 5:** Changes in intestinal microflora after AS/BS intervention

		1st	2nd	3rd
Alfalfa sprout group (*n* = 24)				
Bacteria in stool samples				
	*Bifidobacterium*	11.4 ± 7.80	10.3 ± 8.01	13.4 ± 9.75
	*Lactobacillales*	3.07 ± 3.25	2.19 ± 1.63	3.22 ± 2.69^#^
	*Bacteroides*	49.8 ± 10.9	49.4 ± 9.62	44.4 ± 14.4
	*Prevotella*	2.93 ± 9.45	3.67 ± 9.14	4.22 ± 11.4
	*Clostridium cluster IV*	7.54 ± 4.81	6.23 ± 3.75	6.40 ± 4.72
	*Clostridium subcluster XIVa*	13.7 ± 5.73	13.0 ± 6.31	15.3 ± 6.50
	*Clostridium cluster IX*	3.70 ± 5.09	5.65 ± 6.51	4.77 ± 5.65
	*Clostridium cluster XI*	0.60 ± 1.05	0.63 ± 0.93	0.48 ± 0.60
	*Clostridium cluster XVIII*	1.49 ± 1.83	2.37 ± 3.10	2.61 ± 5.43
	Others	5.83 ± 4.95	6.51 ± 5.55	5.54 ± 2.97

Ammonia (mg/g)		0.644 ± 0.284	0.662 ± 0.319	0.750 ± 0.349

Broccoli sprout group (*n* = 24)				
Bacteria in stool samples				
	*Bifidobacterium*	17.1 ± 9.65	13.1 ± 7.81*	16.6 ± 8.51^#^
	*Lactobacillales*	4.88 ± 7.42	3.06 ± 2.48	4.95 ± 5.34
	*Bacteroides*	42.6 ± 11.1	45.4 ± 14.8	42.6 ± 11.1
	*Prevotella*	2.76 ± 9.19	3.32 ± 9.26	2.76 ± 9.73
	*Clostridium cluster IV*	6.44 ± 4.69	5.90 ± 5.02	5.52 ± 3.81
	*Clostridium subcluster XIVa*	14.0 ± 6.35	14.2 ± 6.29	14.3 ± 5.42
	*Clostridium cluster IX*	6.81 ± 9.23	7.78 ± 9.54	5.96 ± 7.60
	*Clostridium cluster XI*	1.02 ± 1.91	0.90 ± 1.21	1.24 ± 2.63
	*Clostridium cluster XVIII*	1.47 ± 1.55	1.41 ± 1.11	1.23 ± 1.19
	Others	4.38 ± 2.66	5.02 ± 2.97	4.77 ± 2.81

Ammonia (mg/g)		0.645 ± 0.246	0.597 ± 0.251	0.633 ± 0.308

Values are expressed as mean ± SD. *p<0.05: Significant difference from the corresponding value at 1st examination. ^#^*p*<0.05: Significant difference from the corresponding value at 2nd examination.

**Table 6:** Changes in thyroid hormones after AS/BS intervention

	Standard value	1st	2nd	3rd
Alfalfa sprout group (*n* = 24)				
TSH (mIU/ml)	(0.50–5.00)	2.05 ± 1.09	2.34 ± 1.34	2.08 ± 1.27
Free T3 (pg/ml)	(2.30–4.30)	3.04 ± 0.30	3.07 ± 0.30	3.07 ± 0.25
Free T4 (ng/dl)	(0.90–1.70)	1.24 ± 0.17	1.26 ± 0.15	1.25 ± 0.16

Broccoli sprout group (*n* = 24)				
TSH (mIU/ml)	(0.50–5.00)	1.92 ± 1.33	2.13 ± 1.74	2.14 ± 1.91
Free T3 (pg/ml)	(2.30–4.30)	3.25 ± 0.44	2.98 ± 0.45*	3.12 ± 0.51^#^
Free T4 (ng/dl)	(0.90–1.70)	1.28 ± 0.22	1.26 ± 0.22	1.20 ± 0.20^#^

Values are expressed as mean ± SD. *p<0.05: Significant difference from the corresponding value at 1st examination. ^#^*p*<0.05: Significant difference from the corresponding value at 2nd examination.

## Abbreviations

AS: alfalfa sprouts
BMI: body mass index
BS: broccoli sprouts
CSS: constipation scoring system
GI: gastrointestinal
SFN: sulforaphane
SGS: sulforaphane glucosinolate
